# Hydrogen Sulfide Alleviates Oxidative Damage under Chilling Stress through Mitogen-Activated Protein Kinase in Tomato

**DOI:** 10.3390/antiox13030323

**Published:** 2024-03-06

**Authors:** Guoxiu Wu, Xuxu Niu, Jiahui Chen, Changjiang Wu, Yang Li, Yanman Li, Dandan Cui, Xueying He, Fan Wang, Shengli Li

**Affiliations:** 1 College of Horticulture, Henan Agricultural University, Zhengzhou 450046, China; guoxiuwu@henau.edu.cn (G.W.);; 2 Henan Provincial Facility Horticulture Engineering Technology Research Center, Zhengzhou 450046, China; 3 International Joint Laboratory of Henan Horticultural Crop Biology, Zhengzhou 450046, China

**Keywords:** tomato, mitogen-activated protein kinases, chilling stress, oxidative damage

## Abstract

Tomato is the vegetable with the largest greenhouse area in China, and low temperature is one of the main factors affecting tomato growth, yield, and quality. Hydrogen sulfide (H_2_S) plays an important role in regulating plant chilling tolerance, but its downstream cascade reaction and mechanism remain unclear. Mitogen-activated protein kinases (MAPK/MPKs) are closely related to a variety of signaling substances in stress signal transmission. However, whether H_2_S is related to the MPK cascade pathway in response to low-temperature stress is rarely reported. In this study, NaHS treatment significantly decreased the electrolyte leakage (EL), superoxide anion (O_2_^−^) production rate, and hydrogen peroxide (H_2_O_2_) content of seedlings at low temperatures. In addition, the activities of superoxide dismutase (SOD), peroxidase (POD), and catalase (CAT) were obviously increased; and the photochemical efficiency of PSII (Fv/Fm) was enhanced with treatment with NaHS, indicating that NaHS improved the seedlings’ cold tolerance by alleviating the degree of membrane lipid peroxidation and oxidative damage. However, H_2_S scavenger hypotaurine (HT) treatment showed the opposite effect. We found that H_2_S content, L-cysteine desulfhydrase (LCD) activity, and mRNA expression were increased by chilling stress but reduced by MPK inhibitor PD98059; PD98059 reversed the alleviating effect of H_2_S via increasing the EL and H_2_O_2_ contents. The expression levels of *MPK1*–*MPK7* at low temperatures showed that *SlMPK4* was significantly induced by exogenous NaHS and showed a trend of first increasing and then decreasing, while the expression level of *SlMPK4* in HT-treated seedlings was lower than that of the control. After *SlMPK4* was silenced by virus-induced gene silencing, the H_2_S-induced upregulation of C-repeat-Binding Factor (CBF1), inducer of CBF expression 1 (ICE1), respiratory burst oxidase homologs (RBOH1, RBOH2) at low temperatures disappeared, and tomato cold tolerance decreased. In conclusion, H_2_S improves the cold tolerance of tomato plants by increasing the activity of antioxidant enzymes and reducing reactive oxygen species (ROS) accumulation and membrane lipid peroxidation. MPK4 may act as a downstream signaling molecule in this process.

## 1. Introduction

In recent years, chilling stress has become the main factor restricting vegetable yields in agricultural development [1]. Tomato often suffers from low-temperature stress in greenhouse conditions because of its sensitivity to cold, which is the key environmental factor that affects its growth and development and limits its yield and distribution. Therefore, it is of great practical significance and application value to explore a new way to enhance the chilling tolerance of tomato and other high-temperature-loving crops, which can improve crop yield, quality, and economic benefit and guarantee a stable supply of vegetables.

Studies have shown that hydrogen sulfide (H_2_S) is involved in the entire process of plant growth and development and can respond to biotic and abiotic stresses through a variety of signal transduction pathways [2,3]. For example, under salt stress, the synergistic action of Ca^2+^ and H_2_S induced the activity of H^+^-ATPase to form a H^+^ gradient and increase the K^+^/Na^+^ ratio of seedlings, which activated the antioxidant defense system and maintained redox homeostasis and membrane integrity [4]. Under chilling stress, H_2_S as the key upstream component of indoleacetic acid (IAA) could enhance antioxidant capacity, reduce reactive oxygen species (ROS) production, and promote photosynthesis by interacting with nitric oxide (NO), and Ca^2+^, thus improving the chilling tolerance of cucumber [5,6]. The ICE1-CBF-COR (ICE1, inducer of CBF expression; CBF, C-repeat-Binding Factor; COR, cold-responsive) cascade has been widely recognized as the main signal regulation pathway of chilling stress, and there are multiple genes involved in the regulation of chilling stress through *ICE1* and *CBF* transcriptional, translational, and post-translational modification [6]. Zhang et al. [7] demonstrated that H_2_S signal generation induced by low temperatures improved the cold tolerance of cucumber seedlings by upregulating the gene expression levels of *ICE*, *CBF1*, and *COR*. Exogenous H_2_S can improve the stress resistance of grapes by inducing the activity of superoxide dismutase (SOD) and the expression levels of *ICE1* and *CBF3* [8]. These studies indicate that H_2_S can participate in the stress response and signal transmission process of plants in various forms, which is of great significance for alleviating stress, such as low-temperature stress. However, the H_2_S cascade network involved in the tolerance to low-temperature stress in plants is still not entirely clear.

Mitogen-activated protein kinases (MAPK/MPKs) belong to serine/threonine (Ser/Thr) protein kinases, prevalent in eukaryotes, which play important roles in the growth, division, and stress response of plant cells [9]. MPK cascades consist of three kinases, MAPK kinase kinase (MAPKKK), MAPK kinase (MAPKK), and MAPK. When the stress signal is generated outside the cell, the MPK cascades pathway amplifies various stress signals step by step and transmits them to target molecules through consecutive phosphorylation events, thereby causing a series of stress resistance reactions in cells, such as to salt, low temperature, drought, diseases, and insect pests [10,11]. MPKs can mediate physiological activities caused by ethylene, auxin, brassinolide, and other hormones, and play an important role in plant stress resistance [9].

In mammals, H_2_S regulates a variety of physiological processes through the MPK pathway, such as chronic renal failure and gastrointestinal protection [12]. However, there are relatively few studies on the relationship between H_2_S and MPK signaling cascade pathways in plants. MPK6 has a positive effect on the inhibition of primary root growth caused by exogenous H_2_S in *A. thaliana*, accompanied by ROS and NO accumulation [13]. This raises the question of whether H_2_S is related to the MPK cascade pathway in regulating plant cold tolerance. Therefore, this study first identified the responses of H_2_S contents and the MPK cascade to chilling stress in tomato plants, then explored whether MPK was involved in the regulation of H_2_S in cold tolerance, and finally identified the role of MPK4 in H_2_S-induced cold tolerance.

## 2. Materials and Methods

### 2.1. Plant Material and Growth Conditions

The seeds of Ailsa Craig tomato were stored by the factory seedling team of Henan Agricultural University, Zhengzhou, China. Firstly, seeds were disinfected with 2% (*v*/*v*) sodium hypochlorite solution for 15 min, soaked in distilled water for 6 h, then sowed on petri dishes with moist filter paper disks, and finally germinated in the dark at 28 °C. The germinated seeds were sown in an 8 cm × 8 cm nutrient pot with turf, vermiculite, and perlite (*v*/*v*/*v*, 2/1/1) as substrate. The cultivation environment of the climate chamber was as follows: the average daily optical quantum flux density (PFD) was approximately 600 μmol·m^−2^·s^−1^, 26/18 °C (day/night) and 13/11 h (light/dark) photoperiod.

Plants at the three-leaf stage were moved into an artificial climate chamber for the experimental treatment. Dynamic changes in H_2_S and MPK4 signaling were studied at a low temperature of 5 °C for 0 h, 3 h, 6 h, 9 h, 12 h, 24 h, 48 h, and 72 h. To analyze the cold tolerance of the plants, tomato seedlings were treated at normal temperature (25 °C) and low temperature (5 °C) for 48 h. To further study the effect of MPK on the H_2_S signal, tomato seedlings were pretreated with PD98059 (MPK inhibitor) and then placed under chilling stress for 6 h. Exogenous substances were added in the form of 1.0 mmol·L^−1^ NaHS solution, 0.15 mmol·L^−1^ hypotaurine (HT, an H_2_S scavenger), and 0.10 mmol·L^−1^ PD98059 solution; water treatment was used as the control. The exogenous substance was sprayed once a day in the morning for 3 consecutive days, and plants were treated at a low temperature for 24 h after spraying. Each parameter was measured with the same batch of seedlings. Three replicates for each treatment with ten seedlings per replicate were used for further study.

### 2.2. Measurement of H_2_S Content and L-Cysteine Desulfhydrase (LCD) Activity

The H_2_S content was determined using the methylene blue method, followed by fluorescence probe 7-Azido-4-Methylcoumarin (AzMC) staining according to the method of Liu et al. [14], and observed via confocal laser microscopy. A 0.1 g sample of fresh functional leaves was taken, 0.9 mL of 20 mM pre-cooled Tris-HCl buffer (pH 8.0) was added, and the sample was ground into homogenate and centrifuged. The supernatant was collected for the determination of H_2_S content and LCD activity. An absorption well with zinc acetate was placed in a small test tube with the supernatant. After adding 100 μL of 30 mM FeCl_3_ (dissolved in 1.2 M HCl) and 100 μL of 20 mM N, N-dimethyl-p-phenylenediamine (dissolved in 7.2 M HCl), the test tube was quickly sealed with sealing film. The reaction was performed at 37 °C for 30 min. Absorbance was measured at a wavelength of 670 nm.

LCD is one of the main enzymes involved in the generation of H_2_S. The LCD activity was determined by measuring the amount of H_2_S released by L-cysteine (including dithiothreitol) [15].

### 2.3. Antioxidant Enzyme Activity and Electrolyte Leakage (EL)

Samples were prepared by homogenizing the fresh tissue in a solution (4 mL·g^−1^ fresh weight) containing 50 mM KH_2_PO_4_/K_2_HPO_4_ (pH 7.8), 1% PVP, 0.2 mM EDTA, and 1% Triton X-100 with a mortar and pestle. After the homogenate was centrifuged at 12,000× *g* for 20 min at 4 °C, the supernatant was used to determine the enzymatic activities. The malondialdehyde (MDA) content was detected using the method of Heath and Packer [16]. The SOD activity was determined following the Beyer and Fridovich method, and one unit of enzyme activity was expressed by inhibiting 50% of the photochemical reduction [17]. The peroxidase (POD) activity was measured following the method described by Omran, and the activity was expressed by the absorbance change within 1 min at 470 nm [18]. The ascorbate peroxidase (APX) activity was estimated according to the method of Nakano and Asada, and the activity was expressed by the absorbance changes within 1 min of 290 nm [19].

EL was detected following the method presented by Dong et al. [20]. Fresh leaf sample (0.3 g) was soaked at 25 °C in a 15 mL centrifuge tube containing 9 mL deionized water for 6 h, and the electrical conductivity of deionized water (E1) was measured using a portable conductivity meter. Then, the samples were boiled for 20 min, and the electrical conductivity (E2) was measured after cooling to room temperature. Finally, the normal deionized water conductivity (E0) was determined, and the EL was calculated according to the following formula: EL (%) = (E1 − E0)/(E2 − E0) ∗ 100.

### 2.4. Determination of Hydrogen Peroxide (H_2_O_2_) Content and Superoxide Anion (O_2_^−^) Production Rate

The H_2_O_2_ content was determined according to the instructions of H_2_O_2_ Assay Kit A064-1 (Nanjing Jiancheng Bioengineering Institute, Nanjing, China).

The O_2_^−^ production rate was measured using the method of Wang et al. [21]. One gram leaves were ground into a 4 mL solution of 65 mM KH_2_PO_4_/K_2_HPO_4_ (pH 7.8). The supernatant was taken for determination after centrifugation for 15 min. Five hundred microliters of supernatant was shaken well with 500 μL KH_2_PO_4_/K_2_HPO_4_ (50 mM) buffer and 0.1 mL hydroxylamine solution (10 mM) and then kept at 25 °C for 20 min. Then 1 mL p-aminobenzene sulfonic acid (58 mM) and 1 mL naphthylamine (7 mM) were added to the mixed liquor. After 20 min reaction at 25 °C, an equal volume of chloroform was added to extract the pigment and then centrifuged for 3 min. The pink aqueous phase in the upper layer was used for measurement at 530 nm.

### 2.5. Evaluation of Chlorophyll Fluorescence Parameters

After chilling stress, the entire plants were placed in a dark room for 30 min. All indoor light sources were turned off during the measurement to form a dark room environment. The second fully unfolded leaf from top to bottom was collected for image acquisition using the FluorCam 800MF chlorophyll fluorometer (Brno, Czech), and data were recorded.

### 2.6. Virus-Induced Gene Silencing (VIGS)

Primers were designed according to the gene sequence of *SlMPK4* on GenBank. The amplified *SlMPK4* fragments were digested by *Eco*R I/*Bam*H I, and recombined with pTRV2 vector based on tobacco rattle virus (TRV), with empty pTRV2 vector as the control. Furthermore, the pTRV-*PDS* (phytoene desaturase) vector was constructed at the same time. Then, the gene sequence ascertained by sequencing was transformed into the *Agrobacterium tumefaciens* strain GV3101 vector. After 200 seeds were sowed, about 15 days later, tomato seedlings with fully expanded cotyledons were infected 1:1 with *Agrobacterium* carrying pTRV1 and pTRV2 derivatives, respectively. The infected tomato seedlings were cultured in the climate chamber with a temperature of 21 °C and a photoperiod of 12 h for approximately 30 days until the leaves of pTRV-*PDS* plants were bleached. Since PDS is a key enzyme in the pathway of carotenoid synthesis, when the expression of *PDS* is blocked, plants exhibit a whitening effect due to the loss of the photoprotective function of carotenoid. So, it is often used to refer to successful poisoning. The gene silencing efficiency was verified using quantitative real-time polymerase chain reaction (qRT-PCR) as described in Section 2.7, and plants with effective gene silencing were identified. To analyze the effect of H_2_S and MPK4 on H_2_O_2_ signaling in chilling stress, the pTRV- *SlMPK4* seedlings were treated at 5 °C for 6 h, and then sampled using the method in Section 2.1.

### 2.7. qRT-PCR Analysis

The total RNA of the samples was extracted using Trizol and determined using a reverse transcription kit (PrimeScript^®^ RT Master Mix Perfect Real Time) and real-time fluorescence quantitative PCR kit (TB Green^®^ Premix Ex Taq^TM^ II) produced by Takara (Dalian, China). The primers designed using NCBI were synthesized by Shangya Biotechnology (Zhengzhou, China), and the gene expression was measured using BIO-RAD’s real-time fluorescent quantitative PCR instrument, and *EF1a* was used as the reference gene. The relative expression levels were calculated using 2^−△△Ct^. Measurements were performed in triplicate on all of the samples.

The primers’ specificity was compared on the NCBI, and those with better specificity were selected for fluorescence quantification. The specificity of the primers was verified again through the dissolution curve, and those with a unimodal curve with good specificity were selected as the primers.

The primer information is shown in Table 1.

### 2.8. Statistical Analysis

For determinations in all experiments, the data shown in the figures are the mean ± the standard deviation (SD) of three repetitions. Microsoft Excel 2019 software was used for analysis of variance (ANOVA). Duncan’s multiple range test (DMRT) was applied to statistical differences analysis between treatments with the significance level of *p* < 0.05.

## 3. Results and Discussion

### 3.1. Accumulation of H_2_S during Chilling Stress in Tomato Seedlings

**Figure 1:**
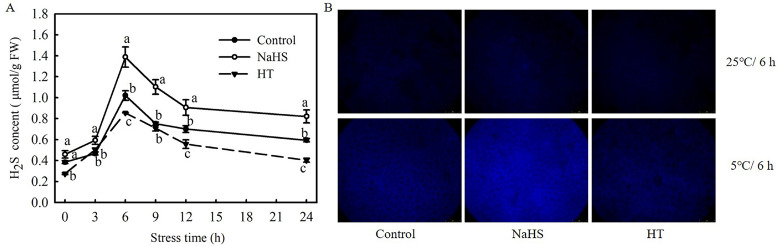
Response of H_2_S signaling to chilling stress and the effect of NaHS on H_2_S accumulation in tomato seedlings. (**A**), H_2_S content; (**B**), H_2_S accumulation fluorescence imaging at 25 °C for 6 h and at 5 °C for 6 h. Three-leaf seedlings were maintained at 5 °C for 24 h. Data shown are the mean ± SD. Different lowercase letters indicate significant differences at *p* < 0.05 among treatments within the same temperature and time.

To investigate the effect of low temperatures on H_2_S formation in tomato seedlings, changes in the H_2_S content in seedling leaves under chilling stress were analyzed. As shown in Figure 1A, with the extension of chilling stress time, the H_2_S content increased during the first 6 h and then decreased promptly within 24 h. The H_2_S content in the NaHS-treated plants was always significantly higher than that in the control plants, while that in the HT-treated plants was significantly lower. H_2_S accumulation was analyzed with a fluorescence microscope, with results in agreement with the biochemical analysis that NaHS treatment induced a stronger fluorescence than the control, and HT content was the least at 6 h chilling stress (Figure 1B). In conclusion, the endogenous H_2_S of tomato seedlings was induced by chilling stress, and exogenously provoked accumulation by NaHS, but was inhibited by HT.

### 3.2. NaHS Mitigated the Oxidative Damage Caused by Chilling Stress through the Regulation of Antioxidant Capacity

**Figure 2:**
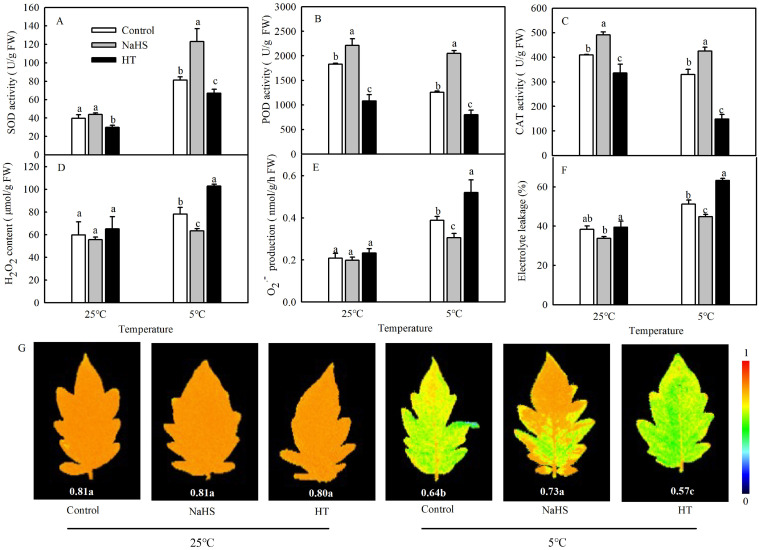
Effects of NaHS on the antioxidant enzyme activities (**A**–**C**); reactive oxygen species (ROS) (**D**,**E**); electrolyte leakage (EL) (**F**); and maximal photochemical efficiency of PSII (Fv/Fm) (**G**) of tomato seedlings under chilling stress. Three-leaf seedlings were maintained at 25 °C and 5 °C for 48 h. Data are the mean ± SD. Different lowercase letters indicate significant differences at *p* < 0.05 among treatments under the same temperature. SOD, superoxide dismutase; POD, peroxidase; and CAT, catalase.

It is known that the production of large amounts of ROS often induces the antioxidant defense system. In order to detect the effects of NaHS on the antioxidant systems of tomato seedlings under chilling stress, the enzyme activities of SOD, POD, and CAT were investigated. As shown in Figure 2A–C, there were some changes in SOD, POD, and CAT activities between the control and NaHS treatment under normal temperatures. Chilling stress significantly increased the SOD activity in the control and decreased POD and CAT activities. Compared with control, NaHS pretreatment increased the activities of SOD, POD, and CAT by 51.8%, 62.7%, and 29.3%, respectively. However, HT treatment significantly decreased the activities of SOD, POD, and CAT under different temperatures. To study the effect of H_2_S induced by NaHS on the cold tolerance of tomato seedlings, the EL, ROS accumulation, and maximal photochemical efficiency of PSII (Fv/Fm) of seedlings were measured. The results showed that the EL, H_2_O_2_ contents and superoxide radical (O_2_^−^) production increased significantly after treatment at 5 °C for 48 h (Figure 2D–F). When the seedlings were pretreated with NaHS, the EL, H_2_O_2_, and O_2_^−^ in the leaves decreased significantly compared with the control under the low temperature but exhibited no difference during exposure to normal temperatures. When HT was applied, the oxidative damage in seedlings caused by chilling stress became serious (Figure 2D-F). Low-temperature treatment at 5 °C for 2 days reduced the Fv/Fm but improved the chilling tolerance, as evidenced by NaHS pretreatment with higher values of Fv/Fm (0.73). The reduction in Fv/Fm was even more serious after H_2_S clearance by HT (Figure 2G).

### 3.3. MPK Inhibitor Decreased the H_2_S Signal and Attenuated the Alleviating Effect of NaHS under Chilling Stress

**Figure 3:**
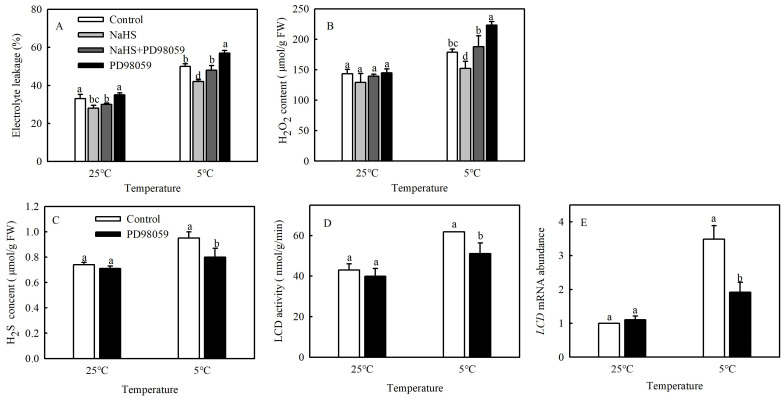
Effects of MPK inhibitor on the alleviating effect of NaHS on chilling stress and H_2_S signaling. (**A**), Electrolyte leakage (EL); (**B**), H_2_O_2_ content; (**C**), H_2_S content; and (**D**,**E**), L-cysteine desulfhydrase (LCD) activity and mRNA expression. Three-leaf seedlings were maintained at 25 °C and 5 °C for 48 h. Data are the mean ± SD. Different lowercase letters indicate significant differences at *p* < 0.05 among treatments under the same temperature.

A large number of studies have shown that MPK cascades can be involved in regulating various stress responses of plants [9]. To explore whether MPK signaling participates in the process of H_2_S alleviating cold damage in tomato, seedlings were pretreated with MPK inhibitor PD98059, and the EL and H_2_O_2_ contents were analyzed. The EL and H_2_O_2_ contents increased dramatically by 14.3% and 23.3%, respectively, after PD98059 was added, compared with NaHS treatment alone at 5 °C. The PD98059-treated seedlings had higher EL and H_2_O_2_ contents (14% and 24.8%, respectively) than the control (Figure 3A,B). The results showed that the alleviation of oxidative damage caused by H_2_S under chilling stress was reversed by the addition of PD98059.

As shown in Figure 3C–E, chilling stress induced higher H_2_S content, LCD activity, and mRNA expression in tomato seedlings. However, PD98059 treatment significantly decreased the H_2_S content, LCD activity, and mRNA expression by 15.8%, 17.3%, and 45.7%, respectively, compared with the control. These findings confirmed that MPK was involved in the production of H_2_S under chilling stress.

### 3.4. SlMPK4 Expression was Increased by NaHS Treatment under Chilling Stress

**Figure 4:**
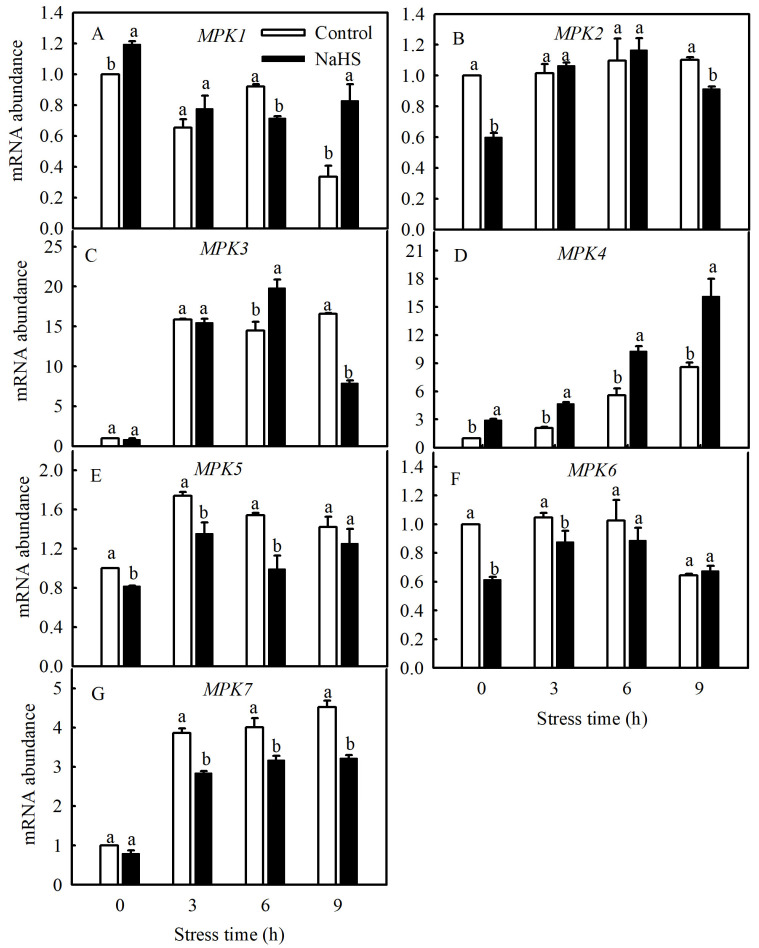
Effects of NaHS on *SlMPKs* expression in tomato seedlings under chilling stress. (**A**–**G**), *MPK1*, *MPK2, MPK3, MPK4, MPK5, MPK6* and *MPK7* expression. Three-leaf seedlings were maintained at 5 °C for 9 h and determined every 3 h. Data are the mean ± SD. Different lowercase letters indicate significant differences at *p* < 0.05 among treatments within the same time.

**Figure 5:**
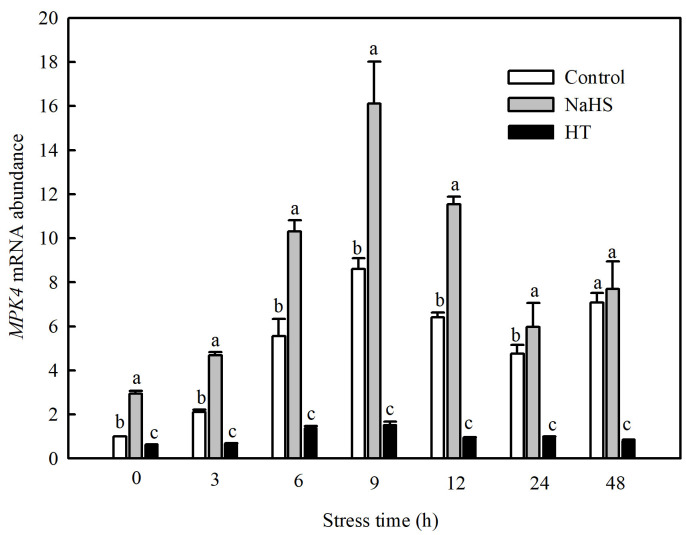
Effects of NaHS on *SlMPK4* expression in tomato seedlings under chilling stress. Three-leaf seedlings were maintained at 5 °C for 48 h and determined at 0 h, 3 h, 6 h, 9 h, 12 h, 24 h, and 48 h. Data are the mean ± SD. Different lowercase letters indicate significant differences at *p* < 0.05 among treatments within the same time. HT, H_2_S scavenger hypotaurine.

Currently, studies have found that the MPK family of tomato consists of 16 members, including *SlMPK1* through *SlMPK7*, which are mainly related to the signal transduction of diseases and abiotic stresses [22]. Therefore, this study investigated the gene expression patterns of *SlMPK1* through *SlMPK7* and the effect of H_2_S on these gene expression patterns at low temperatures. As shown in Figure 4, the expression of *SlMPK3*, *SlMPK4*, *SlMPK5*, and *SlMPK7* increased significantly after chilling stress. In contrast, *SlMPK1* expression decreased, and *SlMPK2* and *SlMPK6* exhibited no obvious change during short periods of exposure to the low temperature. Interestingly, NaHS treatment reduced the expression of *SlMPK5* and *SlMPK7* under chilling stress but increased the expression of *SlMPK4*. Further investigation showed that the low temperature induced the continuous expression of *SlMPK4*, which peaked at 9 h and subsequently decreased but was always higher than that under normal temperatures (Figure 5). The expression level of *SlMPK4* in the NaHS treatment was always higher than that in the control, but the *SlMPK4* expression in the HT treatment was dramatically downregulated (Figure 5).

### 3.5. H_2_S Regulated the Expression of Cold Response-Related Genes and RBOHs via MPK4

**Figure 6:**
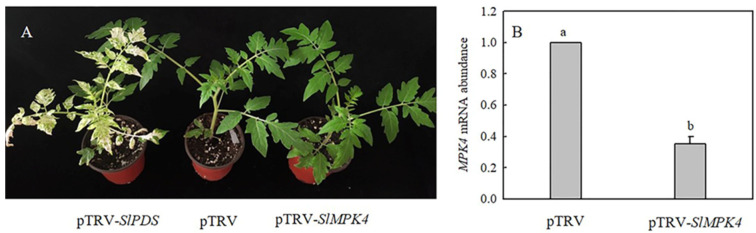
Phenotype of *SlMPK4*-silenced and pTRV-*PDS* plants, and *SlMPK4* gene expression in pTRV-*SlMPK4*. (**A**), Phenotype of tomato 30 days after inoculation with pTRV-*SlPDS* or pTRV-*SlMPK4*. (**B**), Silencing efficiency of *SlMPK4*. The silencing efficiency of *SlMPK4* was measured when plants with pTRV-*SlPDS* turned white. Data are the mean ± SD. Different lowercase letters indicate significant differences at *p* < 0.05.

**Figure 7:**
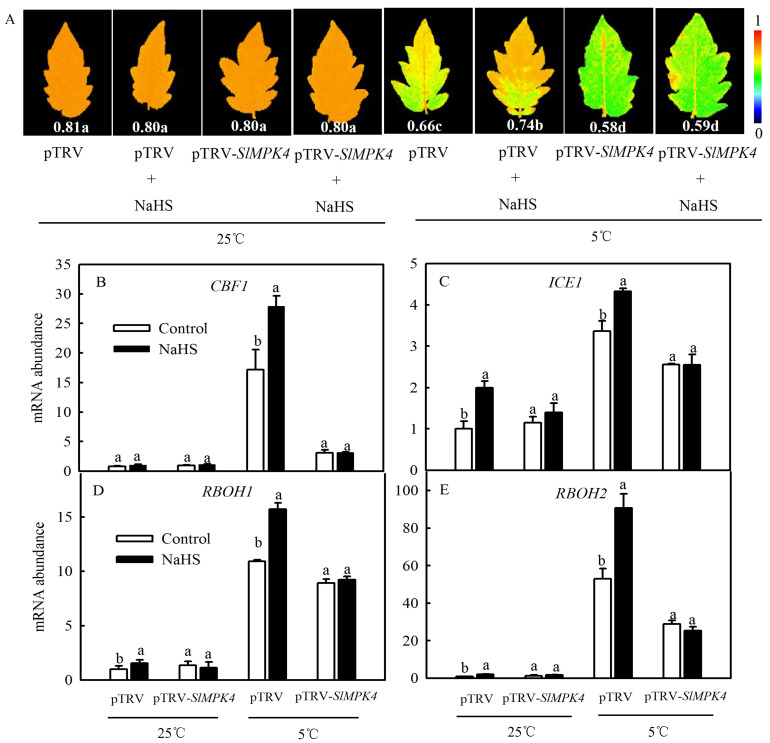
Effects of NaHS on the maximal photochemical efficiency of PSII (Fv/Fm) (**A**); the relative expression of *CBF1* and *ICE1* (**B**,**C**); and the relative expression of *RBOH1* and *RBOH2* (**D**,**E**) in tomato seedlings with *SlMPK4* silencing under chilling stress. (**A**–**C**) were measured when *SlMPK4*-silenced seedlings were maintained at 25 °C and 5 °C for 48 h, while (**D**,**E**) were measured at 25 °C and 5 °C for 6 h. Data are the mean ± SD. Different lowercase letters indicate significant differences at *p* < 0.05 among treatments (control and NaHS) within the same genotype.

To explore the role of MPK4 in H_2_S-induced chilling tolerance in depth, a mature *SlMPK4* VIGS system was established. After 30 days of inoculation, most leaves of pTRV-*PDS* plants showed bleaching, while the control plants grew normally (Figure 6A). Moreover, *SlMPK4* gene expression in the leaves of pTRV-*SlMPK4* plants was reduced by 65% (Figure 6B). Effective *SlMPK4*-silenced plants were then treated at a low temperature. The results showed that silencing *SlMPK4* expression (pTRV-*SlMPK4*) aggravated the sensitivity of plants to chilling stress, as evidenced by the lower Fv/Fm and *CBF1* and *ICE1* expression compared with the empty carrier plants (pTRV). NaHS treatment resulted in higher Fv/Fm and *CBF1* and *ICE1* expression than that in the control of pTRV plants under chilling stress; however, the inductive effect of NaHS disappeared on pTRV-*SlMPK4* plants (Figure 7A–C). Intriguingly, although *SlMPK4* was silenced under chilling stress, both *CBF1* and *ICE1* genes were expressed. These results clearly showed that MPK4 was necessary for H_2_S-induced chilling resistance in tomato plants.

It has been reported that H_2_O_2_ may be involved in H_2_S-induced cold tolerance of cucumber as a downstream signal by enhancing photosynthesis and weakening oxidative damage [23]. We therefore speculated that MPK4 probably plays a role in the regulation of the H_2_S-to-H_2_O_2_ signal. Since RBOH is a key enzyme in the synthesis of H_2_O_2_ in plant, we measured the expression levels of respiratory burst oxidase homolog (*RBOH1*, *RBOH2*) in pTRV-*SlMPK4* or pTRV plants to study the relationship between H_2_S and MPK4 with H_2_O_2_ under chilling stress. As shown in Figure 7D,E, chilling stress significantly induced the expression of either *RBOH1* or *RBOH2*, which was further increased by NaHS. Silencing of *SlMPK4* weakened the transcription of *RBOH1* and *RBOH2* compared with pTRV plants, which were not rescued by the application of NaHS under chilling stress. Meanwhile, chilling stress still could induce the accumulation of *RBOH1* and *RBOH2* transcription in pTRV-*SlMPK4* plants. Accordingly, it was concluded that MPK4 was involved in H_2_S-induced H_2_O_2_ signal bursts in tomato seedlings.

## 4. Discussion

### 4.1. H_2_S Signaling Plays an Important Role in Chilling Stress in Tomato

H_2_S, a colorless gas with the odor of rotten eggs, has long been considered toxic. However, H_2_S now emerges as a novel gas signaling molecule involved in regulating plant growth and development and responding to abiotic stresses [24]. H_2_S is mostly synthesized by LCD and D-cysteine dehydrase (DCD) with L-cysteine and D-cysteine as substrates in plants, of which LCD is the major enzyme that participates in H_2_S production [25]. Generally, various stresses can induce the production of endogenous H_2_S: the present research showed that the endogenous H_2_S level was activated by chilling stress in tomato seedlings. The exogenous H_2_S donor NaHS exogenously provoked H_2_S accumulation in plants, and the H_2_S inhibitor HT decreased the H_2_S content. The changes in endogenous H_2_S levels can influence enzyme activities and gene expressions, and thus modulate plant growth and development. Previous research revealed that with the extension of waterlogging time, the levels of endogenous H_2_S and its main endogenous product cysteine (Cys) increased, and the expression of genes related to H_2_S or Cys biosynthesis and metabolism changed, indicating that H_2_S-Cys homeostasis may have a role in response to waterlogging stress [26].

Many kinds of stresses promote the accumulation of abundant ROS in plants, which is a culprit in oxidative damage. In the present study, chilling stress induced serious oxidative damage in tomato seedlings, and this negative effect could be alleviated by exogenous NaHS and aggravated by HT. Taken together, the findings indicate that H_2_S can maintain ROS homeostasis and membrane integrity in plants through the regulation of antioxidant machinery (enzymes and the AsA-GSH cycle), thereby enhancing plant tolerance to various abiotic stresses.

### 4.2. Relationship between H_2_S and MPK Cascades in Response to Chilling Stress

After the addition of PD98059 or a NO scavenging agent (cPTIO), the effect of H_2_S in cucumber on alleviating nitrate stress was reversed, which revealed that H_2_S probably alleviated the peroxide damage caused by nitrate stress through the MPK/NO signaling pathway [27]. PD98059 could reduce the LCD activity and H_2_S content in tomato seedlings, which indicated that the MPK signaling pathway was involved in the process of H_2_S promoting tomato seedling growth [28]. In the current study, PD98059 pretreatment significantly decreased the endogenous H_2_S content, activity, and relative expression of LCD in chilling stress, compared to the control. Meanwhile, the cold tolerance of plants decreased due to the addition of PD98059. These results demonstrated that the MPK signaling pathway participated in the cold resistance induced by H_2_S in tomato seedlings.

Five MPK genes were found to be significantly induced by exogenous NaHS in tomato roots, namely *MPK3*, *MPK4*, *MPK7*, *MPK11*, and *MPK14* [28]. In the present study, *SlMPK3*, *SlMPK4*, *SlMPK5*, and *SlMPK7* were rapidly induced by a low temperature, and *SlMPK4* expression was continuously induced by NaHS at a low temperature for 9 h and then gradually decreased (Figure 5), indicating that there was a close relationship between H_2_S and MPK4 in regulating the cold tolerance of tomato seedlings. Du et al. [29] used MPK4 mutants in *A. thaliana* as materials and demonstrated that MPK4 is an important downstream component of H_2_S, and both of them help plants resist cold stress via regulating stomatal movement.

At present, most research has been focused on MPK3, MPK4, and MPK6 due to their functions in response to external stimuli. In *A. thaliana*, low-temperature treatment rapidly stimulated the activity of MPK3, MPK4, and MPK6. MPK3/6 is upstream of ICE1 and negatively regulated its cold tolerance through the phosphorylation of ICE1 [30]. Zhao et al. [31] considered that the MEKK1-MKK2-MPK4 pathway suppressed MPK3 and MPK6 activities and had a positive role in the cold response, which reduced the degradation of ICE1 and activated the expression of *CBF* and *COR* genes. In other words, MPK4 plays a positive role in the regulation of plant cold signaling and enhances the freezing resistance of plants. In the present study, H_2_S induced the expression of *CBF1* and *ICE1* at a low temperature, but this induction disappeared after *SlMPK4* silencing (Figure 7B,C), which indicated the crucial role of MPK4 in H_2_S-induced cold resistance. Meanwhile, we found that the expression of *CBF1* and *ICE1* could be detected in *SlMPK4*-silenced seedlings under chilling stress, but it was weaker than that in the control plants. Thus, we speculated that the MPK4 pathway is not essential in the cold induction of *CBFs.*

ROS include H_2_O_2_, O_2_^−^, hydroxyl radical (·OH), singlet oxygen (1O_2_), etc. Their production plays an important role in response to stress and regulation of plant development within a certain controllable range. H_2_O_2_ is the main form of ROS and interacts with other signals. When ROS content is too high, oxygen metabolism will be unbalanced, causing degeneration of biological macromolecules such as DNA, proteins, and lipids. Therefore, the balance of H_2_O_2_ content is very important for mitigating the damage of stress [32]. The interaction between H_2_S and ROS is both relative and synergistic [33].

H_2_S can reduce ROS accumulation by increasing the activities of antioxidant enzymes such as CAT and glutathione reductase (GR) to reduce oxidative damage caused by various stresses [14]. The MPK signaling pathway acts downstream of receptor-like protein kinases and ROS signaling to regulate ROS-related gene expression and programmed cell death [34]. The heat tolerance of CRISPR/Cas9-*SlMPK3* plants was significantly higher than that of the wild type, indicating that *SlMPK3* negatively regulated the heat tolerance of tomato and played an important role by regulating ROS production and scavenging [35]. However, in the present study, some evidence to explain the mechanism by which MPK4 decreases ROS accumulation through antioxidant enzymes under chilling stress is required. Oxidative damage caused by low temperatures and other stresses is closely related to ROS metabolism. H_2_S and MPK can alleviate biotic or abiotic stresses by regulating ROS balance. What is the relationship between the ROS balancing and the H_2_S-dependent *MPK4* expression to regulate plant cold tolerance? Long-term chilling stress promoted an increase in the levels of ROS (Figure 2D,E). Therefore, and as a response, H_2_S activates protective systems such as those of SOD, POD, and CAT to eliminate ROS (Figure 2A–C). However, under short-term chilling stress, there was an induction of *RBOH1* and *RBOH2* by H_2_S with further production of H_2_O_2_, and in this case with signaling function. Moreover, the inductive effect disappeared after silencing *SlMPK4* (Figure 7D,E). However, there was more *RBOH1* and *RBOH2* expression under chilling stress in pTRV-*SlMPK4* plants compared with normal temperature. But, the *RBOH1* and *RBOH2* expressions of pTRV-*SlMPK4* plants were lower than those of pTRV plants. The results indicated that there might be another pathway independent of MPK4 in response to ROS signaling induced by chilling stress. Therefore, this work preliminarily speculated that H_2_S might affect the ROS balance through MPK4 to regulate the cold tolerance of tomato, and the more detailed mechanism needs to be further explored.

## 5. Conclusions

**Figure 8:**
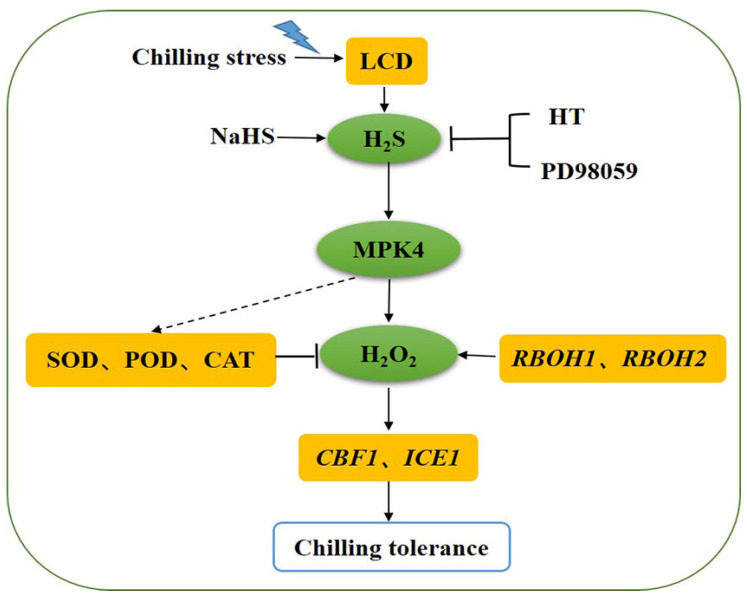
Model of the possible relationship between H_2_S and MPK4 in tomato seedlings under chilling stress. Both H_2_S signaling and *SlMPK4* expression are rapidly activated in chilling stress, and exogenous H_2_S can upregulate *SlMPK4* expression. H_2_S from NaHS can improve chilling tolerance via increasing antioxidant enzyme activities and decreasing reactive oxygen species (ROS) accumulation, and these effects are reversed by the addition of H_2_S scavenger and MPK inhibitor PD98059. The H_2_S-triggered upregulation of *CBF1* and *ICE1* disappears when *SlMPK4* is silenced under chilling stress, as does the *RBOH1* and *RBOH2* expression required for H_2_O_2_ production. Therefore, MPK4 may act downstream of the H_2_S signaling involved in chilling tolerance induced by H_2_S. However, the direct mechanism of action of MPK4 on SOD, POD, CAT needs to be further explored. SOD, superoxide dismutase; POD, peroxidase; CAT, catalase; HT, H_2_S scavenger hypotaurine; and LCD, L-cysteine desulfhydrase.

LCD-dependent H_2_S could protect tomato seedlings from the oxidative damage caused by chilling stress through regulating the antioxidant enzyme activities and ROS balance. MPK4 was found to be essential for the promotion of the expression of *RBOH1* and *RBOH2* by H_2_S that produced the H_2_O_2_ signal. The study preliminarily established a signaling pathway in which MPK4 could possibly act downstream of the H_2_S signaling and participate in H_2_S-induced chilling tolerance. Meanwhile, MPK4 may play an essential role in cold tolerance by regulating the H_2_S-induced ROS balance (Figure 8). But, the further mechanisms between H_2_S and MPK4 need to be better explored.

## Figures and Tables

**Table 1:** Primer sequences.

Gene	Accession No.	Primer Name (5′→3′)	Primer Position	Amplicon Size (bp)
*EF1a*	NM_001247106	F:5′-ATTGGAAACGGATATGCCCCT-3′ R:5′-TCCTTACCTGAACGCCTGTCA-3′	1106–1226	101
*LCD*	XM_026030768	F:5′-CATTTTGCGGTGGAAGTCCC-3′ R:5′-TAGTCGTTTGGCCCCATCTG-3′	1337–1558	222
*ICE1*	NM_001287789	F:5′-GGAGCACAACCAACCCTTTT-3′ R:5′-TTCCCCACTACCCCACTTTC-3′	857–1009	153
*CBF1*	NM_001247194	F:5′-TCTGATTCTGCTTGGAGGCT-3′ R:5′-AAGATCGCCTCCTCATCCAC-3′	353–525	173
*RBOH1*	NM_001374505	F: 5′-CCGGGAGCAAGGATCATTTG-3′ R: 5′-AAGTGCCTGGACCATGGTAA-3′	2625–2778	154
*RBOH2*	NM_001247342	F: 5′-TATGGGTCCCTGTGTGTGTC-3′ R: 5′-TGTATTGCAACCCCAAGTGC-3′	1437–1637	201
*MPK1*	NM_001247082	F: 5′-CGCTCTTGCACATCCTTACC-3′ R: 5′-ACCTGCAACAATCTGTCAGC-3′	1044–1238	195
*MPK2*	NM_001247426	F: 5′-CAAGATGGTACCGACCTCCA-3′ R: 5′-AAGCAGACGTAGCTGGTGTA-3′	757–908	152
*MPK3*	NM_001247431	F: 5′- ATGTTTGGTCTGTGGGTTGC -3′ R: 5′- GGGAGTTGCCTGACGTATCT -3′	800–974	175
*MPK4*	NM_001246838	F: 5′-GTTCACCGGAGGAGTCTGAT-3′ R: 5′-GGGACACATCAGGGAAATGC-3′	792–908	117
*MPK5*	NM_001247337	F: 5′-TCTCCTGGAGCGGTTGATTT-3′ R: 5′-ATGGAGAGGTGCCAAGTAGG-3′	1056–1160	105
*MPK6*	NM_001247731	F: 5′-TGGTCAGTGGGTTGCATACT-3′ R: 5′-TTGCCTTGGGTACCTTGGAA-3′	956–1141	186
*MPK7*	NM_001246968	F: 5′-ATGTCCGACAGCTTCCTCAA-3′ R: 5′-TAATTCGACGGGTTGGGTCA-3′	922–1041	120

## Data Availability

Data are contained within the article.
